# Antifungal and Antiaflatoxigenic Potential of Lactic Acid Bacteria Against *Aspergillus flavus* and *Aspergillus parasiticus* for Wheat Grain Protection

**DOI:** 10.3390/microorganisms14081710

**Published:** 2026-08-04

**Authors:** Mohammed Aladhadh, Fatma Ibrahim Abou-Elazm, Rushdy M. Ahmed, Rafaat M. Elsanhoty, Mahmoud A. Al-Saman, Samar S. Mabrouk

**Affiliations:** 1 Department of Food Science and Human Nutrition, College of Agriculture and Food, Qassim University, Buraydah 51452, Saudi Arabia; 2 Department of Microbiology and Immunology, Faculty of Pharmacy, Misr University for Science and Technology, Giza 32361, Egypt; fatma.alsayed@must.edu.eg; 3 Special Food and Nutrition Department, Food Technology Research Institute, Agriculture Research Center, Giza 32361, Egypt; rushdy.m@yahoo.com; 4 Department of Industrial Biotechnology, Faculty of Biotechnology (FBT), University of Sadat City (USC), Sadat City 22857, Egypt; rafaat.elsanhoty@gebri.usc.edu.eg; 5 Microbiologyand Immunology Department, Faculty of Pharmacy, Ahram Canadian University (ACU), 6th October City 12566, Egypt; samar.mabrouk@acu.edu.eg

**Keywords:** lactic acid bacteria, *Lactobacillus plantarum*, *Lactobacillus acidophilus*, *Aspergillus flavus*, *Aspergillus parasiticus*, aflatoxin, biological control, wheat grains, fungi

## Abstract

Aflatoxin contamination of cereal grains represents a serious food safety concern due to the toxic and carcinogenic properties of aflatoxins produced mainly by *Aspergillus* species. The present study aimed to evaluate the antifungal and antiaflatoxigenic potential of lactic acid bacteria (LAB) cell-free supernatants as a natural approach for controlling aflatoxin-producing fungi in wheat grains. A total of fifty fungal isolates recovered from agricultural samples were screened for their aflatoxigenic potential using phenotypic and molecular approaches. The toxigenic isolates were identified as *Aspergillus flavus* (AF) and *Aspergillus parasiticus* (AP), and their aflatoxigenic potential was confirmed by PCR amplification of aflatoxin biosynthesis-related genes (*nor-1* and *aflR*). The antifungal activity of selected LAB strains was evaluated against the identified *Aspergillus* isolates using the agar diffusion method. The obtained results demonstrated that LAB cell-free supernatants exhibited significant antifungal activity, with variations among strains. Among the tested LAB strains, *Lactobacillus plantarum* P3 and *Lactobacillus acidophilus* ATCC 20552 showed the highest inhibitory activity against both fungal species. Furthermore, LAB treatments significantly reduced fungal-induced wheat grain damage and decreased aflatoxin accumulation during storage. Application of 100% LAB supernatants resulted in a remarkable reduction in total aflatoxins, reaching more than 99% reduction compared with untreated controls. The inhibitory effect decreased with increasing dilution of the supernatants, indicating a concentration-dependent antifungal and antiaflatoxigenic activity. The findings demonstrate that LAB-derived metabolites can effectively suppress the growth of aflatoxin-producing *Aspergillus* species and limit aflatoxin biosynthesis. Therefore, LAB cell-free supernatants represent a promising biological control strategy for improving cereal safety and reducing mycotoxin contamination in food systems.

## 1. Introduction

Aflatoxins (AFs) are hazardous secondary metabolites mainly produced by several *Aspergillus* species, including *A. flavus*, *A. parasiticus*, and the less frequently reported *A. nomius* [1,2]. The contamination of food and feed commodities with aflatoxins represents a major global concern due to their toxic, carcinogenic, and mutagenic effects, leading to serious health risks and considerable economic losses [3,4,5]. Among the naturally occurring aflatoxins, B_1_, B_2_, G_1_, and G_2_ are the most prevalent, with aflatoxin B_1_ considered the most potent hepatocarcinogen in animals and a major threat to food safety [6,7]. Lactic acid bacteria (LAB) are among the most effective beneficial microorganisms exhibiting antimicrobial activities. Their inhibitory effects are attributed to several mechanisms, including rapid nutrient competition, environmental acidification, and the production of a wide range of antimicrobial compounds during carbohydrate metabolism [8,9]. In addition to organic acids, LAB can synthesize various bioactive metabolites, such as bacteriocins (e.g., nisin), reuterin, and reutericyclin, which contribute to their antagonistic activity against undesirable microorganisms [8,10]. The increasing demand for safe and natural food preservation strategies has stimulated interest in LAB-derived antimicrobials as potential bio-preservatives [11,12]. Recent studies have demonstrated that LAB possess antifungal properties and can inhibit the growth of food-associated molds and yeasts [9].

Several LAB species, including *Lactobacillus pentosus* [13], *L. rhamnosus* [14,15], *L. salivarius*, *L. sanfrancisco* [16,17], and *L. plantarum* [18,19,20] have been reported to exhibit antifungal activity. The first investigations on the antifungal properties of lactobacilli and streptococci were conducted by El-Gendy and Marth [21], who suggested that proteinaceous antifungal compounds contributed significantly to LAB-mediated fungal inhibition. Furthermore, some LAB strains, such as *L. plantarum* and *L. sanfrancisco*, produce specific organic acids with antifungal properties, including 3-phenyllactic acid and caproic acid [17,19,22,23]. Mycotoxin contamination remains a significant challenge in cereal production, with approximately 25% of global grain production estimated to be affected annually, resulting in substantial economic losses worldwide [24]. Therefore, the development of effective and environmentally friendly approaches to reduce fungal contamination and prevent mycotoxin production during grain storage is highly important. Biological preservation using LAB represents a promising alternative due to their safety, compatibility with food systems, and ability to produce antifungal metabolites.

However, the efficiency of LAB strains in controlling aflatoxigenic *Aspergillus* species varies considerably depending on the strain, fungal species, and the nature of produced antifungal metabolites. Therefore, further investigation of LAB isolates with antifungal and antiaflatoxigenic activities is still required. In this context, the present study aimed to evaluate the antifungal and antiaflatoxigenic potential of fifteen LAB strains against aflatoxin-producing *A. flavus* and *A. parasiticus*. The selected LAB strains were screened for antifungal activity using in vitro assays, while preliminary tests were performed to exclude possible inhibitory effects caused by the growth medium or acidity of the supernatants. The most effective LAB strains were further evaluated for their ability to reduce fungal growth, wheat grain deterioration, and aflatoxin accumulation during storage. The study provides insight into the potential application of LAB as natural biocontrol agents for improving wheat grain safety and reducing aflatoxin contamination.

## 2. Materials and Methods

### 2.1. Characterization of Lactic Acid Bacterial Strains

Table 1 summarizes the characteristics of the LAB strains used in the present study, including their source, optimum growth temperature, and oxygen requirements. A total of fifteen LAB strains belonging mainly to the genus *Lactobacillus*, in addition to *Streptococcus thermophilus* and *Bifidobacterium animalis* subsp. *lactis*, were evaluated for their antifungal potential against aflatoxin-producing *Aspergillus* species. The tested strains were obtained from different microbial culture collections and research institutions. Most LAB strains exhibited facultative anaerobic or aerotolerant characteristics, which are typical features of LAB, allowing their growth under limited oxygen conditions. The diversity of the tested strains provides a suitable basis for screening their inhibitory activity and identifying promising candidates for fungal control and wheat protection. The identity of the obtained LAB strains was confirmed based on the information provided by the respective microbial culture collections.

### 2.2. Maintenance and Preservation of LAB Strains

The collected LAB strains were cultivated on de Man, Rogosa, and Sharpe (MRS) agar medium (Difco Laboratories, Sparks, MD, USA) to ensure optimal growth and maintenance. Anaerobic and oxygen-sensitive strains were incubated and maintained under anaerobic conditions using Anaerogen sachets in Oxoid anaerobic jars. After sufficient growth, the developed colonies were preserved on MRS agar plates at 4 °C for short-term storage and were sub-cultured monthly to maintain viability and purity. For long-term preservation, cell suspensions were prepared and stored in cryovials at −80 °C using 20% glycerol as a cryoprotective agent until further experimental use.

### 2.3. Isolation and Identification of Fungal Strains

The *A. flavus* and *A. parasiticus* strains used in this study were isolated from different cereal grains, including wheat, corn, and barley. Samples were subjected to mycological analysis by plating unsterilized cereal grains onto Potato Dextrose Agar (PDA; Oxoid, Hampshire, UK) supplemented with streptomycin (50 mg/L) to suppress bacterial growth. Approximately 10–15 grains were placed on each PDA plate using sterile forceps, and the plates were incubated at 25 ± 2 °C for 5–7 days [25]. Emerging fungal colonies were purified by repeated sub-culturing on fresh PDA plates. The purified isolates were identified and confirmed by the Fungal Taxonomy Department, Institute of Plant Pathology, Agricultural Research Center (ARC), Giza, Egypt. Prior to use, the fungal cultures were maintained on PDA slants at 5 °C and sub-cultured monthly to preserve their viability [26].

### 2.4. Pre-Screening Tests and Evaluation of Potential Antifungal Compounds

The antifungal activity of concentrated and pasteurized MRS medium was initially evaluated using the agar diffusion assay, as previously described by Zhao et al. [20]. To investigate the potential effect of pH on antifungal activity, MRS medium was concentrated ten-fold and adjusted to pH values ranging from 1.0 to 6.0 before pasteurization. This experiment was conducted separately to assess the contribution of pH to the observed antifungal activity.

Each LAB strain was cultivated in liquid MRS broth under its optimal growth conditions with respect to temperature and oxygen availability. The LAB strains were cultivated under static conditions, consistent with their facultative anaerobic nature. After 5 days of incubation, the bacterial cultures were centrifuged at 6000× *g* for 15 min at 4 °C to obtain cell-free supernatants containing potentially produced antifungal metabolites. The collected supernatants were diluted with deionized distilled water to obtain concentrations equivalent to 50 and 25% of the original supernatant. The different concentrations were pasteurized at 70 °C for 20 min and stored at 4 °C until further evaluation of their antifungal activity using the agar diffusion assay. When required, the pH of the concentrated supernatants was adjusted using sterile NaOH or HCl solutions before pasteurization. All experiments were performed in triplicate, and the results were expressed as mean ± standard deviation.

### 2.5. Assay of Antifungal Activity

The antifungal activity of LAB strains was evaluated in vitro using the disc diffusion method according to De Muynack et al. [26], with some modifications. The preparation and cultivation of the fungal strains and the preparation of fungal spore suspensions were performed as described in Section 2.8, with the spore suspension adjusted to approximately 7 × 10^7^ spores/mL. Briefly, sterile Whatman No. 1 filter paper discs (5 mm diameter) were placed on PDA plates previously inoculated with 100 µL of fungal spore suspension. Subsequently, 10 µL of the prepared LAB-derived antifungal solution or cell-free supernatant was applied to each disc. For each fungal strain, discs without LAB-derived antifungal solution or cell-free supernatant were used as positive controls to represent fungal growth in the absence of LAB treatment, whereas uninoculated PDA plates were used as negative controls. The inoculated plates were incubated at 25 °C for 5 days, and antifungal activity was determined by measuring the diameter of the clear inhibition zones formed around the discs (mm). The inhibition zone values were expressed as the mean of three independent replicates. The antifungal effectiveness of LAB metabolites was interpreted according to the diameter of the inhibition zones. Inhibition zones of ≤8 mm were considered indicative of insensitivity or low antifungal activity, zones of 9–14 mm indicated sensitivity, zones of 15–19 mm indicated very high sensitivity, and zones of ≥20 mm indicated extremely high sensitivity.

### 2.6. Screening of Aflatoxin-Producing Isolates of A. flavusand A. parasiticus

#### 2.6.1. Fluorescence Technique

The aflatoxin-producing ability of *A. flavus* and *A. parasiticus* isolates was initially screened using the fluorescence technique described by Yabe et al. [27]. A total of fifty fungal isolates were tested for their potential aflatoxin production. The fungal isolates were inoculated at different points on Czapek-Dox Agar (CDA) plates and incubated for seven days at 25 ± 2 °C. Non-fluorescent glass Petri dishes were used to facilitate the detection of aflatoxin-related fluorescence. After incubation, the plates were examined under ultraviolet (UV) light at 365 nm, where the diffusible aflatoxin compounds appeared as fluorescent zones surrounding the fungal growth. Based on the positive fluorescence screening results and subsequent PCR confirmation, one *A. flavus* isolate (AF) and one *A. parasiticus* isolate (AP) were selected for subsequent challenge experiments.

#### 2.6.2. Molecular Technique

##### Sample Preparation

The selected *A. flavus* and *A. parasiticus* isolates, categorized according to their aflatoxin-producing ability based on the fluorescence assay (high, moderate, and non-producers), were prepared for molecular analysis. Ten 250 mL Erlenmeyer flasks containing 100 mL of Czapek-Dox Broth medium (Merck KGaA, Darmstadt, Germany) were sterilized by autoclaving at 121 °C for 20 min. Each flask was inoculated with one 5-mm-diameter mycelial disc excised from the actively growing margin of a 7-day-old fungal colony on PDA using a sterile cork borer and incubated for 7 days at 25 ± 2 °C. After incubation, the fungal mycelial mats were collected by centrifugation at 10,000× *g* for 10 min and stored at 4 °C until DNA extraction [28].

##### DNA Isolation and Extraction

Genomic DNA extraction was performed using a modified protocol based on the method described by Yelton et al. [29]. The collected fungal mycelia from each isolate were transferred into a mortar, frozen with liquid nitrogen, and ground into a fine powder. The powdered mycelial material was suspended in lysis buffer containing 50 mM/L EDTA and 0.2% SDS (pH 8.5), followed by incubation at 68 °C for 15 min. After centrifugation at 15,000× *g* for 20 min, the supernatant was transferred to a new centrifuge tube, and sodium acetate (2 mM/L) was added. The mixture was incubated on ice for 1 h and centrifuged again at 15,000× *g* for 15 min. The recovered supernatant was transferred to a fresh tube, and phenol extraction was performed to remove contaminants. Finally, DNA precipitation was achieved by adding 2.5 volumes of ethanol, and the extracted DNA was collected for further molecular analysis.

#### 2.6.3. PCR-Based Assay

PCR amplification was performed according to Farber et al. [30] with some modifications. The extracted genomic DNA from the selected *A. flavus* and *A. parasiticus* isolates was used as a template for amplification of aflatoxin biosynthesis-related genes.

The DNA samples were adjusted to a concentration of 2 µg/mL before PCR amplification. PCR reactions were carried out in a final volume of 50 µL using the reaction mixture components presented in Table 2. The amplification program consisted of an initial denaturation step at 95 °C for 1 min, followed by 35 cycles of denaturation at 94 °C for 30 s, annealing at 65 °C for 30 s, and extension at 72 °C for 30 s. Specific primers targeting the aflatoxin biosynthesis genes nor-1and aflR-1were used for molecular detection of aflatoxin-producing isolates (Table 3). The amplified PCR products were separated by electrophoresis on a 2% agarose gel using 1× TBE buffer (45 mM boric acid, 40 mM Tris-Cl, and 1 mM EDTA; pH 8.3) at 75 V for 3 h. Following ethidium bromide staining, the amplified fragments were visualized under a UV transilluminator (Thermo Fisher Scientific, Waltham, MA, USA). The sizes of the amplified products were determined using a 1 kb DNA ladder marker (used as a molecular size marker, New England Biolabs, Ipswich, MA, USA). The presence of nor-1and aflR-1genes was confirmed by detection of specific amplicons with sizes of 367 bp and 400 bp, respectively [31].

### 2.7. Preparation of Wheat Kernel Samples

Wheat grains were prepared according to Osman [34] with some modifications. Briefly, the wheat grains were thoroughly washed with sterile distilled water and dried in a hot-air oven at 44 °C for 42 h. The dried grains were mixed with sterilized sand for 1 min to facilitate surface cleaning before being treated with a 5% sodium hypochlorite solution for 2 min, and then rinsed three times with sterile distilled water. The moisture content of the wheat grains was determined using a grain moisture analyzer at the Central Laboratory for Food and Feed, Agriculture Research Center (ARC), Giza, Egypt. After preparation, the samples were separated into sub-samples and transferred into sterile 1.0 L plastic containers according to Eisa et al. [35] until further experimental use.

### 2.8. Inoculation of Spore Suspension and Treatment of Wheat Kernels

Spore suspensions of *A. flavus* and *A. parasiticus* were prepared from 21-day-old pure cultures grown on 9 cm PDA plates. The fungal cultures were flooded with 15 mL of sterile distilled water and gently scraped for 1–2 min to release the spores. The obtained suspensions were filtered through three layers of sterile cheesecloth to remove mycelial fragments. The spore concentration was determined using a Spencer hemocytometer and adjusted to approximately 7 × 10^7^ spores/mL. The prepared spore suspension was used to inoculate wheat grains to obtain a final concentration of approximately 10^3^ spores/g grains according to Eisa et al. [35]. The prepared wheat grain samples were divided into three main groups: (i) uninoculated grains used as a negative control, (ii) grains inoculated with *A. flavus* spores, and (iii) grains inoculated with *A. parasiticus* spores. The moisture content of all treatments was adjusted to 20% according to the American Association of Cereal Chemists [36] by adding calculated volumes of the following solutions:

I. Sterile distilled water only (positive control without LAB treatment).
II. 75% sterile distilled water + 25% LAB cell-free supernatant.
III. 50% sterile distilled water + 50% LAB cell-free supernatant.
IV. 100% LAB cell-free supernatant.

For each treatment, 100 wheat kernels were used as one experimental unit (replicate), and each treatment was performed using three independently prepared and treated containers. The required volume of each treatment solution was calculated based on the initial moisture content of the wheat grains to achieve a final moisture content of 20%. The containers were incubated at 25 ± 2 °C for 30 days. Independent containers were assigned to each sampling time (0, 15, and 30 days), and each sampling time was therefore represented by independent biological replicates. Individual kernels within each container were considered subsamples and were not treated as independent biological replicates.

The above treatments were applied using the selected LAB strains (*L. acidophilus* ATCC 20552 and *L. plantarum P3*). All treated samples were incubated at room temperature for 30 days. After the incubation period, the percentage of grain damage and aflatoxin content were determined. For each fungal inoculation group, wheat kernels were treated with different concentrations of LAB cell-free supernatant. Each treatment was performed in triplicate, with each replicate consisting of 100 wheat kernels.

### 2.9. Determination of Total Grain Damage Percentage

The percentage of total grain damage was determined by counting the number of infected kernels in three independent replicates, each consisting of 100 wheat kernels. The damage percentage was calculated based on the number of visibly infected or damaged kernels after the incubation period.

### 2.10. Determination of Aflatoxin Levels

#### 2.10.1. Extraction and Cleanup Procedure

The extraction and cleanup procedures were performed at the Pesticides Research Laboratories, Etay El-Baroud Research Station, Agriculture Research Center (ARC), Giza, Egypt, according to Choochuay et al. [37], with modifications. Briefly, 10 g of finely ground wheat grains were mixed with 20 mL of acetonitrile:methanol (40:60, *v*/*v*), allowed to stand for 30 min at 25 °C, and then shaken for an additional 30 min. Subsequently, 1 g of sodium chloride (NaCl) and 4 g of magnesium sulfate (MgSO_4_) were added, and the mixture was shaken for 10 min. An aliquot (2 mL) of the supernatant was evaporated to dryness and reconstituted with 0.5 mL of 10% acetonitrile. The derivatization was performed using trifluoroacetic acid (TFAA), followed by incubation for 15 min. After cooling, the prepared samples were subjected to liquid chromatographic analysis. All chemicals were supplied by El Nasr for Pharmaceutical Chemicals (ADWIC), Cairo, Egypt.

#### 2.10.2. High-Performance Liquid Chromatography (HPLC)

Aflatoxins, B_1_, B_2_, G_1_, and G_2_ were quantified at the Central Laboratory, Faculty of Agriculture, Alexandria University, Egypt, using an Agilent Technologies 1260 Infinity HPLC system equipped with a fluorescence detector (Agilent Technologies, Santa Clara, CA, USA). Chromatographic separation was performed using a C_18_ analytical column (3.9 × 25 mm, 5 µm particle size) maintained at 40 °C. The analysis was conducted at a flow rate of 1 mL/min using an isocratic mobile phase consisting of acetonitrile:methanol:water (15:15:70, *v*/*v*/*v*) for 20 min. Detection was performed at excitation and emission wavelengths of 360 and 440 nm, respectively. The chromatograms of B_1_, B_2_, G_1_, and G_2_ were identified and quantified by comparison with corresponding aflatoxin standards using ChemStation software, version C.01.07 (Santa Clara, CA, USA). Representative HPLC chromatograms of the aflatoxin standards and a wheat grain sample are presented in Supplementary Figure S1A,B.

#### 2.10.3. Method Validation and Quality Control

Fortified aflatoxin-free wheat grain samples were analyzed during method development to evaluate method performance, including recovery and limits of detection (LODs). For recovery assessment, aflatoxin standards were added to aflatoxin-free wheat grain samples at two concentration levels before the extraction step, and the spiked samples were analyzed using the procedure described above. All analyses were performed in duplicate under the same experimental conditions. The LOD was determined as the lowest aflatoxin concentration producing a response three times greater than the average baseline noise obtained from 10 independent blank samples. The analytical method and instruments were validated as part of the laboratory quality assurance system, and the relevant Codex Committee criteria for quality assurance were followed to assess the performance of the method for aflatoxin determination.

### 2.11. Statistical Analysis

All experiments were conducted using three independent experimental replicates, and the results are presented as standard error of the mean (SEM). The reported replicates (*n* = 3) corresponded to independent biological replicates rather than repeated analytical or technical measurements. The experimental unit for each assay is defined in the corresponding Materials and Methods subsection. One-way ANOVA was performed separately for each experiment, and statistical comparisons were conducted only among the experimental treatments within the corresponding assay according to the available experimental design. Data were statistically analyzed using one-way analysis of variance (ANOVA), followed by Tukey’s honestly significant difference (HSD) post hoc test at *p* ≤ 0.05. The statistical analysis was performed according to the methods described by Gomez and Gomez [38].

## 3. Results and Discussion

### 3.1. Fungal Isolation and Identification

A total of 92 fungal isolates were recovered from different food and agricultural samples suspected to be contaminated with fungi. The obtained isolates were screened and identified based on their macro- and microscopic morphological characteristics. Suspected *Aspergillus* isolates were further screened for their aflatoxin-producing ability using UV fluorescence (365 nm) and ammonium vapor tests. Aflatoxin-producing isolates showed characteristic blue fluorescence under long-wave UV illumination and developed a pink coloration after exposure to ammonium fumes. The selected aflatoxigenic isolates were further confirmed by molecular analysis using gene-specific PCR targeting aflatoxin biosynthesis-related genes. Based on morphological, biochemical, and molecular characterization, two representative isolates were selected and designated as AF and AP isolates, corresponding to *A. flavus* and *A. parasiticus*, respectively. After 10 days of growth on PDA medium, the AF isolate exhibited typical characteristics of *A. flavus*, producing yellowish-green colonies with a diameter of approximately 6–8 cm. The AP isolate was identified as *A. parasiticus*. The identification of the fungal isolates was performed according to the standard taxonomic keys described by Samson et al. [39].

### 3.2. Identification of Aflatoxigenic Aspergillus Species

**Figure 1:**
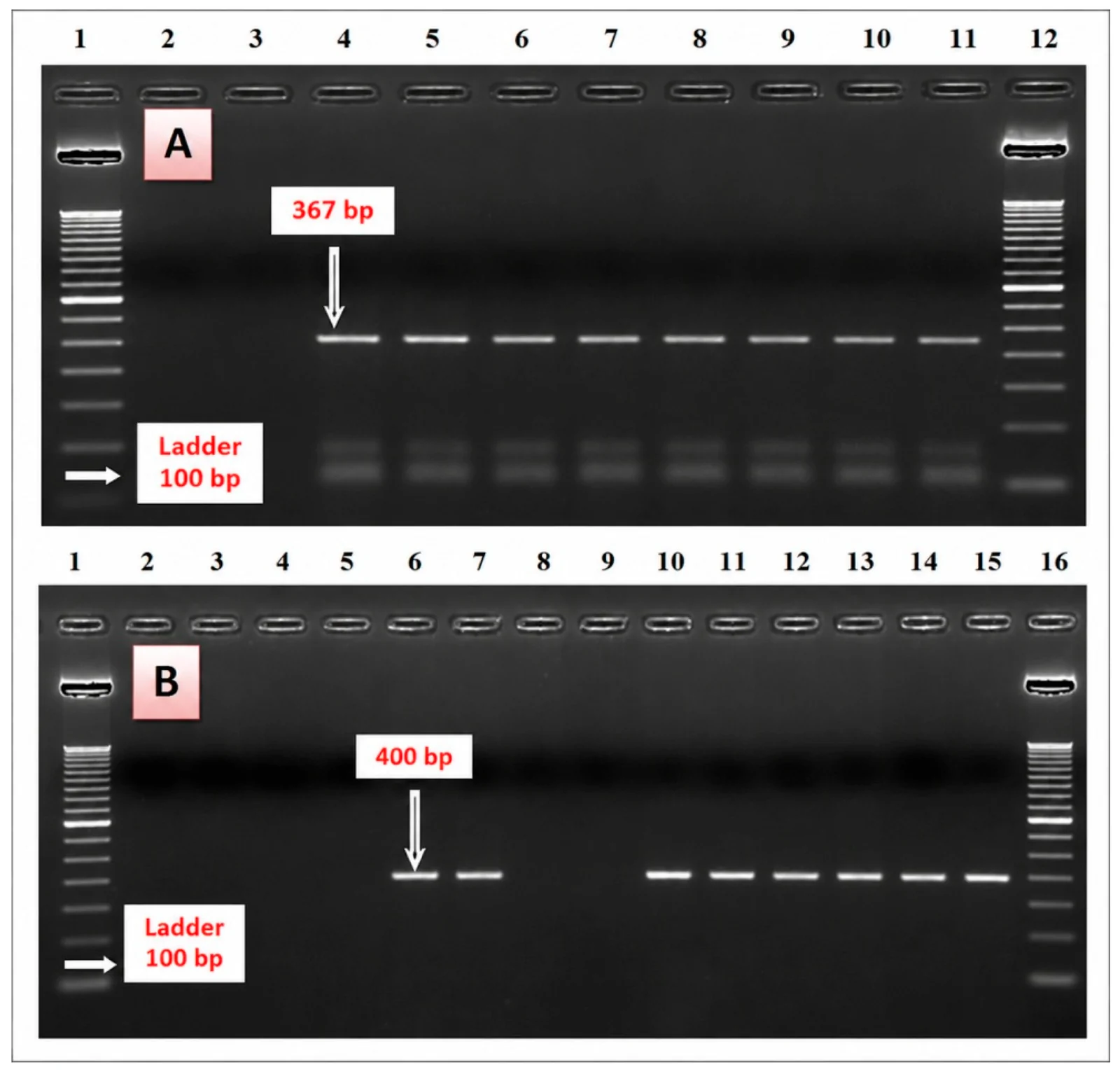
PCR amplification of aflatoxin biosynthesis-related genes in selected *Aspergillus* isolates. (**A**) Amplification of the nor-1 gene showing the expected 367 bp amplicon. (**B**) Amplification of the aflR gene showing the expected 400 bp amplicon. PCR products were visualized using a 100 bp DNA ladder.

The genomic DNA of the selected fungal isolates was extracted using the phenol–chloroform–isoamyl alcohol method and used for molecular confirmation of the aflatoxigenic *Aspergillus* isolates. Following morphological identification and preliminary screening for aflatoxin production using fluorescence-based methods, the selected *A. flavus* (AF) and *A. parasiticus* (AP) isolates were further confirmed by PCR amplification of aflatoxin biosynthesis-related genes. The presence of the aflatoxin-associated genes *nor-1* and aflRwas detected using gene-specific primers listed in Table 3. PCR amplification revealed the expected specific amplicons for both target genes. The *nor-1* gene produced an amplified fragment of approximately 367 bp (Figure 1A), whereas the *aflR* gene generated an amplicon of approximately 400 bp (Figure 1B) compared with the 100 bp DNA ladder. The detection of both *nor-1* and *aflR* genes in the selected AF and AP isolates confirmed their potential aflatoxin-producing capability and supported the morphological and fluorescence-based identification of *A. flavus* and *A. parasiticus* isolates used in the subsequent experiments.

### 3.3. Pre-Screening Tests

A preliminary screening experiment was performed to determine whether the antifungal activity observed from LAB culture supernatants could be attributed to MRS medium components or acidity effects. The antifungal activity of concentrated and pasteurized MRS medium was evaluated using the agar diffusion assay. No inhibition zones were observed around the filter discs containing concentrated MRS medium, indicating that the MRS components themselves did not exhibit antifungal activity against the tested *Aspergillus* strains. Furthermore, the effect of pH on fungal growth inhibition was evaluated using concentrated MRS medium adjusted to different pH values ranging from 1.0 to 6.0. The results indicated that when the pH value was above 3.0, no significant antifungal inhibition was observed; suggesting that acidity alone was not responsible for the antifungal effect. Although *A. flavus* and *A. parasiticus* showed some sensitivity under highly acidic conditions, the observed antifungal activity of LAB-derived supernatants could not be attributed solely to low pH. These findings confirm that the antifungal effects detected in subsequent experiments were mainly related to bioactive compounds produced by LAB rather than the acidity of the supernatants or MRS medium components. Similar observations were reported by De Muynck et al. [26], who demonstrated that MRS medium and supernatant acidity do not exhibit antifungal activity when the pH conditions are above the inhibitory range.

### 3.4. Antifungal Activity of LAB Cell-Free Supernatants Against Aflatoxigenic Aspergillus Species

The antifungal activity of the tested LAB strains was evaluated using the disc diffusion method against the aflatoxin-producing fungi *A. flavus* and *A. parasiticus* (Table 4). The concentrated and pasteurized cell-free supernatants obtained from LAB cultures exhibited variable inhibitory effects against the tested fungal strains. These findings indicate that the antifungal activity of LAB is strain-dependent and varies according to the ability of each strain to produce antifungal metabolites. The results showed that most of the tested LAB strains exhibited antifungal activity, while some strains showed weak or no inhibitory effect against the indicator fungi. Among the tested strains, *L. plantarum* P3 showed the highest inhibitory activity, producing inhibition radii of 20 and 18 mm against *A. flavus* and *A. parasiticus*, respectively. In addition, *L. acidophilus* ATCC 20552 showed considerable antifungal activity with inhibition zones of 21 and 17 mm against *A. flavus* and *A. parasiticus*, respectively.

Statistical analysis confirmed significant differences among the tested LAB strains in their antifungal activity against both *A. flavus* and *A. parasiticus* (*p* ≤ 0.05). The inhibition zones varied significantly among strains, confirming a strain-dependent antifungal effect. *L. acidophilus* ATCC 20552 and *L. plantarum* P3 showed the strongest inhibitory activity against both fungal species, whereas *L. salivarius* TISTR 390 showed no detectable inhibition. Based on the inhibition-zone classification, *L. acidophilus* ATCC 20552 and *L. plantarum* P3 were classified as extremely sensitive against *A. flavus*, whereas *L. plantarum* P3 was classified as very sensitive against *A. parasiticus*. The remaining LAB strains showed varying degrees of sensitivity, ranging from low or no antifungal activity to sensitive and very sensitive responses.

These strains were therefore considered promising candidates for further application in wheat protection experiments. The observed antifungal activity of LAB has been previously reported and may be attributed to the production of different bioactive compounds, including organic acids, bacteriocin-like substances, phenyllactic acid, 4-hydroxyphenyllactic acid, short-chain fatty acids, and other low-molecular-weight antimicrobial compounds [13,40]. These metabolites can interfere with fungal growth by affecting cellular metabolism, reducing mycelial development, and limiting fungal sporulation [41]. The inhibitory effect differed between the two tested fungal species, with *A. parasiticus* showing higher susceptibility to some LAB supernatants compared with *A. flavus*. Such variation may be related to differences in fungal physiology and sensitivity toward the antimicrobial metabolites produced by LAB. Similar differences in fungal inhibition by LAB-derived compounds have been reported previously [42]. The reduction in antifungal activity observed with lower concentrations of LAB supernatants (if applied in subsequent dilution experiments) may be associated with the decreased concentration of active antifungal compounds, as reported for *L. acidophilus* supernatants where dilution resulted in reduced inhibitory activity against fungal strains [43,44]. The obtained results confirm the potential of selected LAB strains, particularly *L. acidophilus* ATCC 20552 and *L. plantarum P3,* as effective biological control agents against aflatoxin-producing *Aspergillus* species. Similar antifungal properties of LAB against food-associated fungi and mycotoxin-producing microorganisms have been described in previous studies [20,45,46].

### 3.5. Effect of LAB Cell-Free Supernatants on Wheat Grain Protection Against Aflatoxigenic Aspergillus Species

The protective effect of LAB cell-free supernatants against fungal deterioration of wheat grains was evaluated by determining the percentage of damaged kernels during a 30-day storage period. The results presented in Table 5 demonstrated that treatment with LAB supernatants reduced the percentage of grain damage compared with the corresponding positive controls inoculated with *A. flavus* or *A. parasiticus*. The protective effect varied depending on the LAB strain, fungal species, and supernatant concentration. In uninoculated wheat grains (negative control), the damage percentage remained relatively low during storage. After 30 days, untreated grains showed 8% damage for samples treated with *L. acidophilus* ATCC 20552 and 9% for samples treated with *L. plantarum* P3 at 100% concentration, indicating that LAB supernatants did not adversely affect grain quality.

In contrast, inoculation with *A. flavus* caused a marked increase in grain damage. The positive control reached 100% damage after 30 days for *L. acidophilus* and *L. plantarum* experiments, respectively. Treatment with LAB supernatants significantly reduced fungal damage. The highest protection was observed with 100% supernatant treatment, where damage percentages were reduced to 26 and 17% for *L. acidophilus* ATCC 20552 and *L. plantarum P3*, respectively. Lower concentrations showed reduced protective activity, with damage increasing as the supernatant concentration decreased. A similar trend was observed in wheat grains inoculated with *A. parasiticus*. The untreated positive control exhibited the highest damage percentage (100%) after 30 days. Application of LAB supernatants reduced grain deterioration, particularly with the 100% treatment, which resulted in damage percentages of 24% for *L. acidophilus* ATCC 20552 and 22% for *L. plantarum P3*. However, dilution of the supernatants decreased their protective effect, resulting in higher damage percentages. The obtained results indicate that LAB-derived metabolites can suppress fungal development and delay grain deterioration during storage. The stronger protective effect observed with undiluted supernatants suggests that the antifungal compounds produced by LAB act in a concentration-dependent manner. This finding agrees with previous observations that LAB strains can produce antifungal metabolites capable of limiting fungal growth and reducing food spoilage caused by mycotoxin-producing fungi. *L. plantarum* P3 and *L. acidophilus* ATCC 20552 showed promising protective effects against both *A. flavus* and *A. parasiticus*, supporting their potential application as biological control agents for reducing fungal damage in wheat grains.

The reduction in the total damage percentage of wheat grains treated with LAB cell-free supernatants may be attributed to the ability of LAB-derived metabolites to inhibit the mycelial growth and development of different *Aspergillus* species, thereby limiting fungal colonization and subsequent sporulation. Similar observations were reported by Onilude et al. [41] and Elsanhoty [18], who suggested that the antifungal compounds produced by LAB can suppress fungal growth and reduce the deterioration of food commodities caused by toxigenic fungi.

The protective effect of LAB cell-free supernatants was further evaluated by calculating the percentage reduction in wheat grain damage compared with untreated fungal controls. The results demonstrated that both *L. acidophilus* ATCC 20552 and *L. plantarum* P3 exhibited considerable protective activity against damage caused by aflatoxin-producing *Aspergillus* species. The highest reduction in grain damage was recorded for *L. plantarum* P3 against *A. flavus* (83%), followed by *L. plantarum* against *A. parasiticus* (78%). Similarly, *L. acidophilus* ATCC 20552 showed reduction percentages of 74 and 76% against *A. flavus* and *A. parasiticus*, respectively. These findings indicate that LAB-derived metabolites can effectively limit fungal development and reduce grain deterioration during storage. The higher protective effect observed for *L. plantarum* may be related to its greater ability to produce antifungal compounds capable of inhibiting fungal growth and colonization. Similar antifungal and bio-protective effects of LAB against food-associated fungi have been previously reported [20]. The mean total grain damage across the three storage periods was lower in samples treated with higher concentrations of LAB supernatants. For *L. acidophilus* ATCC 20552, the mean damage values were 6.00, 11.33, and 20.00% for the 100, 50, and 25% supernatant treatments, respectively, in the negative controls. In *A. flavus*-infected grains, the corresponding mean damage values increased to 14.00–60.67%, while *A. parasiticus*-infected grains showed mean damage values ranging from 14.33 to 65.00%. A similar trend was observed for *L. plantarum* P3, where increasing the concentration of the LAB supernatant was associated with lower mean grain damage across the storage period. These descriptive results indicate that higher concentrations of LAB supernatants were associated with reduced grain damage during storage, with the lowest damage observed following treatment with 100% supernatant.

### 3.6. Effect of LAB Cell-Free Supernatant on Aflatoxin Accumulation in Wheat Grains

The effect of *L. plantarum* P3 cell-free supernatant on aflatoxin accumulation in wheat grains inoculated with aflatoxin-producing *Aspergillus* species was evaluated by determining the concentrations of aflatoxins B_1_, B_2_, G_1_, and G_2_ (Table 6). In wheat grains inoculated with *A. flavus*, the untreated control (0% LAB supernatant) exhibited the highest total aflatoxin concentration (74,260 ppb). Treatment with 100% *L. plantarum* P3 supernatant markedly reduced the total aflatoxin concentration to 121 ppb. The 50% and 25% supernatant treatments resulted in total aflatoxin concentrations of 22,361 and 19,994 ppb, respectively. Similarly, in wheat grains inoculated with *A. parasiticus*, the untreated control showed a total aflatoxin concentration of 34,834 ppb, whereas treatment with 100% *L. plantarum* P3 supernatant reduced the total aflatoxin concentration to 102 ppb. The 50% and 25% supernatant treatments resulted in total aflatoxin concentrations of 16,115 and 17,304 ppb, respectively. The 100% *L. plantarum* P3 supernatant exhibited the greatest reduction in total aflatoxin accumulation for both fungal species.

The effect of *L. acidophilus* ATCC 20552 cell-free supernatant was also evaluated (Table 6). In wheat grains inoculated with *A. flavus*, the untreated control showed the highest total aflatoxin concentration (146,280 ppb), whereas treatment with 100% *L. acidophilus* ATCC 20552 supernatant reduced the total aflatoxin concentration to 139 ppb. The 50% and 25% supernatant treatments resulted in total aflatoxin concentrations of 559 and 9507 ppb, respectively. In wheat grains inoculated with *A. parasiticus*, the untreated control exhibited a total aflatoxin concentration of 34,820 ppb, which decreased to 143 ppb following treatment with 100% *L. acidophilus* ATCC 20552 supernatant. The 50% and 25% supernatant treatments resulted in total aflatoxin concentrations of 11,555 and 25,790 ppb, respectively. These findings demonstrate that the undiluted cell-free supernatants of both LAB strains substantially reduced aflatoxin accumulation compared with the untreated fungal controls, although the magnitude of reduction varied depending on the LAB strain, fungal species, and supernatant concentration.

The protective efficiency of LAB treatments was evaluated by calculating the percentage reduction in wheat grain damage and total aflatoxin accumulation compared with untreated fungal controls (Table 7). Both *L. plantarum* P3 and *L. acidophilus* ATCC 20552 exhibited strong protective effects against aflatoxigenic *Aspergillus* species. The highest reduction in grain damage was observed for *L. plantarum* P3 against *A. flavus* (83%), whereas the lowest damage reduction was recorded for *L. acidophilus* ATCC 20552 against *A. flavus* (74%). Regarding aflatoxin accumulation, both LAB strains showed remarkable antiaflatoxigenic activity, with reduction percentages exceeding 99% for both *A. flavus* and *A. parasiticus*. The higher reduction in aflatoxin levels compared with grain damage reduction indicates that LAB metabolites may not only suppress fungal growth but may also interfere with aflatoxin biosynthetic pathways. These findings support the potential use of LAB as biological control agents for reducing fungal deterioration and aflatoxin contamination in wheat grains.

The concentrations of individual aflatoxins (B_1_, B_2_, G_1_, and G_2_) and total aflatoxins were evaluated in wheat grains after treatment with LAB cell-free supernatants. The results demonstrated that both *L. plantarum* P3 and *L. acidophilus* ATCC 20552 markedly reduced aflatoxin accumulation after 30 days of storage compared with untreated fungal controls. The highest aflatoxin concentrations were detected in wheat grains infected with *A. flavus* and *A. parasiticus* without LAB treatment, confirming their strong aflatoxigenic potential. In contrast, wheat grains treated with 100% LAB supernatants showed the lowest levels of individual and total aflatoxins, indicating the strong antiaflatoxigenic activity of LAB-derived metabolites.

Among the detected aflatoxins, B_1_ was generally one of the predominant toxins across the tested treatments, followed by varying proportions of G_1_, B_2_, and G_2_. This finding is consistent with the well-established ability of aflatoxigenic *Aspergillus* species to produce B_1_ as a major and highly toxic aflatoxin. The observed reduction in aflatoxin accumulation may be attributed to the inhibitory effects of LAB metabolites on fungal growth and/or their potential interference with aflatoxin biosynthetic pathways.

The obtained results revealed that the reduction in aflatoxin accumulation was dependent on the concentration of LAB cell-free supernatants. As the concentration of the supernatant decreased, aflatoxin production increased, indicating that dilution reduced the antifungal and antiaflatoxigenic activity of the LAB-derived metabolites. Similar observations were reported by Batish and Lal [47] and Plockova et al. [43,44], who demonstrated that dilution of LAB supernatants resulted in a reduction in their inhibitory activity against fungal growth. Comparable findings have also been reported by Ghonaimy et al. [48], Abouloifa et al. [28], Sehar et al. [49], and Cui et al. [50]. In the present study, *A. parasiticus* showed higher production of aflatoxins G_1_ and G_2_ compared with *A. flavus* following treatment with *L. acidophilus* ATCC 20552 supernatant, suggesting differences in aflatoxin biosynthetic profiles between the two fungal species. Moreover, treatment with *L. plantarum* cell-free supernatant resulted in a marked reduction in aflatoxin accumulation after 30 days of storage, confirming its potential inhibitory effect against aflatoxigenic fungi. The comparative results indicated that both *L. plantarum* P3 and *L. acidophilus* ATCC 20552 exhibited strong antiaflatoxigenic activity; however, the magnitude of inhibition varied depending on the fungal species and LAB strain. The antifungal activity of LAB may be associated with the production of organic acids, bacteriocin-like compounds, and other antimicrobial metabolites capable of inhibiting fungal growth and interfering with aflatoxin biosynthesis.

Due to their importance in various food fermentation processes, LAB have attracted considerable attention as potential biological control agents [51]. Previous studies have demonstrated the antifungal capability of LAB strains against toxigenic fungi. For example, *L. casei* was reported to inhibit the growth and aflatoxin production of *A. parasiticus* [18,21,49,50]. Additionally, Vanne et al. [52] reported that LAB could inhibit the growth of storage fungi, while Onilude et al. [41] demonstrated the ability of *L. plantarum* to suppress the growth of *A. flavus*. These effects were suggested to be related to a combination of microbial competition, acidity, and production of antifungal metabolites [53].

The screening results demonstrated that LAB possess considerable antifungal potential, highlighting their promising role as natural biopreservatives for controlling food spoilage fungi. Most of the tested LAB strains exhibited antifungal activity against aflatoxin-producing *Aspergillus* species, suggesting their potential application in reducing fungal contamination and improving food safety. The antifungal activity of LAB cannot be attributed only to the reduction in pH resulting from organic acid production; rather, it may involve the production and secretion of different bioactive metabolites with antifungal properties. Organic acids can enhance antifungal activity by creating unfavorable conditions for fungal growth, while other antimicrobial compounds may directly affect fungal cell structures and metabolic processes [54,55]. Previous studies have demonstrated that LAB strains can produce various antifungal compounds, including proteinaceous substances, bacteriocin-like peptides, and low molecular weight metabolites. For example, antifungal activity associated with proteinaceous compounds was reported for *L. acidophilus* strains [18,42,47]. Additionally, several studies have indicated that LAB can produce metabolites capable of inhibiting filamentous fungi and yeasts [26,41,49,56].

The variation in antifungal efficiency among LAB strains may be related to differences in their ability to synthesize and release inhibitory compounds. This could explain the differences observed in the present study between *L. plantarum* P3 and *L. acidophilus* ATCC 20552 against *A. flavus* and *A. parasiticus*. Therefore, the antifungal effect of LAB appears to result from a synergistic action of acidification, antimicrobial metabolites, and strain-specific mechanisms rather than a single inhibitory factor.

The reduction in aflatoxin accumulation observed after LAB treatments may be attributed to several proposed mechanisms involved in mycotoxin removal and detoxification. One of the suggested mechanisms is the adsorption of mycotoxins onto the cell wall components of LAB, which facilitates their removal from contaminated food matrices. This binding ability has been associated with the structural components of LAB cell walls, including polysaccharides, proteins, and peptidoglycans [57]. Wang et al. [58] reported that selected LAB strains were able to reduce patulin levels in culture media through binding activity, which was enhanced in strains exhibiting higher specific surface area and greater cell wall volume due to the increased availability of binding sites. The authors suggested that polysaccharides and proteins represent the major functional components involved in mycotoxin adsorption. Furthermore, the binding efficiency of mycotoxins by LAB cells may be influenced by several factors, including the initial mycotoxin concentration, LAB cell density, bacterial strain specificity, food matrix composition, pH, and incubation temperature [59]. Dalié et al. [60] demonstrated that viable cells are not always essential for mycotoxin binding, as aflatoxin B_1_ can interact with specific components present in the LAB cell wall. In addition to adsorption, another possible mechanism involves the interaction between mycotoxins and bioactive metabolites produced by LAB. These metabolites, including organic acids, phenolic compounds, fatty acids, reuterin, and low molecular weight bioactive peptides, may contribute to mycotoxin reduction by binding, modifying, or reducing the toxic effects of these compounds [61]. Although the exact mechanisms responsible for LAB-mediated mycotoxin reduction remain incompletely understood, previous studies have suggested three major pathways: enzymatic degradation of mycotoxins by LAB-derived enzymes, adsorption of mycotoxins by bacterial cell wall components, and interactions between mycotoxins and LAB-produced metabolites. Therefore, the strong reduction in aflatoxin levels observed in the present study following treatment with *L. plantarum* P3 and *L. acidophilus* ATCC 20552 may be related not only to fungal growth inhibition but also to potential binding and detoxification mechanisms mediated by LAB cells and their bioactive metabolites.

A limitation of the present study is that the LAB cell-free supernatants were not normalized according to bacterial biomass (e.g., OD_600_ or CFU/mL) before collection. Therefore, the 100, 50, and 25% supernatant concentrations represent relative dilution levels rather than standardized doses of antifungal metabolites, and differences in antifungal activity among LAB strains may partly reflect differences in metabolite production and accumulation during growth under the applied conditions. In addition, although a separate pH-dependent experiment using MRS medium adjusted to different pH values was performed to investigate the potential contribution of acidity, pH-matched controls corresponding to the actual pH of individual LAB supernatants were not included; thus, the specific contribution of acidity relative to other LAB-derived antifungal metabolites cannot be completely distinguished. Furthermore, the experimental data were not analyzed using a multi-factorial statistical model to simultaneously assess the main effects and interactions among fungal species, LAB strains, supernatant concentration, and incubation time. The statistical analyses were performed according to the individual experimental comparisons and the available experimental structure. Future studies should therefore incorporate standardized bacterial biomass before supernatant collection, appropriate pH-matched controls, and a factorial experimental design with multi-factorial statistical modeling to better characterize strain-specific antifungal activity and comprehensively evaluate the main effects and interactions among these experimental factors. In addition, although aflatoxin analysis was performed using a validated HPLC method in an accredited analytical laboratory, numerical analytical validation parameters (e.g., recovery, limit of detection, limit of quantification, and analytical precision) were not available from the laboratory records and therefore could not be reported. Future studies should include these analytical performance characteristics to further strengthen the methodological transparency and reproducibility of the analysis.

## 4. Conclusions

The present study demonstrated the promising antifungal and antiaflatoxigenic potential of LAB cell-free supernatants against aflatoxin-producing *A. flavus* and *A. parasiticus*. Molecular identification confirmed the presence of aflatoxin biosynthesis-related genes (*nor-1* and *aflR*) in the selected fungal isolates, confirming their toxigenic potential. The evaluated LAB strains, particularly *L. plantarum* P3 and *L. acidophilus* ATCC 20552, exhibited strong inhibitory effects against fungal growth and significantly reduced wheat grain deterioration and aflatoxin accumulation during storage. The remarkable reduction in total aflatoxin levels following LAB treatment indicates that LAB-derived metabolites possess both antifungal and antiaflatoxigenic activities, which may involve inhibition of fungal development and interference with aflatoxin biosynthetic pathways. The concentration-dependent effect of LAB supernatants highlights the importance of bioactive compound levels in achieving effective fungal control. The findings support the potential application of LAB cell-free supernatants as safe and natural biocontrol agents for reducing fungal contamination and improving the safety and quality of cereal grains. Further studies focusing on the identification of specific antifungal metabolites and their mechanisms of action are recommended to enhance their application in food preservation systems.

## Figures and Tables

**Table 1:** Growth temperature, oxygen requirement, and source of lactic acid bacterial strains used in the study.

#	Strains	Source	Growth Temperature (°C)	Oxygen Requirement
1	*Lactobacillus acidophilus* ATCC 20552	3	35–37	Microaerophilic/ aerotolerant anaerobe
2	*Lactobacillus casei* TISTR 453	2	30–37	Facultatively anaerobic/ aerotolerant anaerobe
3	*Lactobacillus rhamnosus* TISTR 541	2	37	Facultatively anaerobic/aerotolerant anaerobe
4	*Lactobacillus helveticus* LH-BO2	2	37–42	Facultatively anaerobic/ aerotolerant anaerobe
5	*Lactobacillus delbrueckii subsp. bulgaricus* EMCC3	1	42–45	Microaerophilic/ aerotolerant anaerobe
6	*Lactobacillus johnsonii* ATCC 3320	3	37	Microaerophilic/ aerotolerant anaerobe
7	*Streptococcus thermophilus* EMCC-2	1	42	Facultatively anaerobic
8	*Lactobacillus acidophilus* ATCC 4356	3	37	Microaerophilic/ aerotolerant anaerobe
9	*Lactobacillus acidophilus* P2	5	37	Microaerophilic/ aerotolerant anaerobe
10	*Lactobacillus plantarum* P3	5	30–37	Facultatively anaerobic/aerotolerant anaerobe
11	*Lactobacillus acidophilus* P4	5	37	Microaerophilic/ aerotolerant anaerobe
12	*Lactobacillus buchneri* P1	5	30	Facultatively anaerobic/aerotolerant anaerobe
13	*Lactobacillus brevis* ATCC 8287	3	30	Facultatively anaerobic/aerotolerant anaerobe
14	*Lactobacillus salivarius* TISTR 390	2	37	Facultatively anaerobic/aerotolerant anaerobe
15	*Bifidobacterium animalis* subsp. *lactis* (DSMZ)	4	37–40	Anaerobic (oxygen-sensitive)

1. Egyptian Microbial Culture Collection (EMCC), Cairo Microbiological Resources Centre (Cairo MIRCEN), Faculty of Agriculture, Ain Shams University, Cairo, Egypt. 2. Thailand Institute of Scientific and Technological Research (TISTR), Bangkok, Thailand. 3. The American Type Culture Collection (ATCC), Manassas, VA, USA. 4. Deutsche Sammlung von Mikroorganismen und Zellkulturen GmbH (DSMZ), Germany. 5. The P1 to P4 strains were obtained from University of Sadat City, Faculty of Biotechnology, Industrial Biotechnology Department, Food and Dairy Biotechnology Branch, Egypt, through collaboration with Prof. Dr. Hoda Mahrous (these strains were not obtained from a formal culture collection, and therefore do not have official collection numbers).

**Table 2:** Composition of the PCR reaction mixture used for amplification of aflatoxin biosynthesis-related genes.

Chemical	Volume (μL)
Primers (2 μM/μL)	8.0
10× buffer	5.0
2 mM dNTP Mix	5.0
Taq DNA polymerase (5 U/μL)	0.5
Template DNA (50 ng)	2.0
Sterile distilled water	29.5
Total	50.0

**Table 3:** Primer sequences used for amplification of aflatoxin biosynthesis-related genes in *Aspergillus* isolates.

Gene	Primer	Sequence (5′-3′)	Size (bp)	Ref.
*nor-1*	nor1 F	TTGGCCGCCAGCTTCGACA	367	Setlem and Ramlal [32]
	nor1 R	TCGCTGAAGGCAGTCGGAT		
*aflR*	aflR F	GCACCTGTCTTCCCTAACA	400	Priyanka et al. [33]
	aflR R	ACGAACATGCTCAGCAAGTA		

**Table 4:** Antifungal activity of cell-free supernatants produced by LAB strains against aflatoxin-producing *A. flavus* and *A. parasiticus* as determined by the agar diffusion assay.

LAB Strains	Aflatoxin-Producing Test Fungi	
	*A. flavus* AF	*A. parasiticus* AP
*L. acidophilus* ATCC 20552	21.00 ± 0.404 ^a^	17.00 ± 0.346 ^ab^
*L. casei* TISTR 453	10.00 ± 0.260 ^e^	7.00 ± 0.144 ^g^
*L. rhamnosus* TISTR 541	13.00 ± 0.173 ^cd^	8.00 ± 0.231 ^g^
*L. helveticus* LH-BO2	14.00 ± 0.289 ^c^	10.00 ± 0.173 ^f^
*L. delbrueckii* subsp. *bulgaricus* EMCC3	8.00 ± 0.375 ^f^	5.00 ± 0.121 ^h^
*L. johnsonii* ATCC 33200	12.00 ± 0.462 ^d^	12.00 ± 0.214 ^de^
*S. thermophilus* EMCC-2	14.00 ± 0.144 ^c^	11.00 ± 0.260 ^ef^
*L. acidophilus* ATCC4356	13.00 ± 0.173 ^cd^	13.00 ± 0.144 ^cd^
*L. acidophilus* P 2	16.00 ± 0.260 ^b^	16.00 ± 0.115 ^b^
*L. plantarum* P3	20.00 ± 0.115 ^a^	18.00 ± 0.346 ^a^
*L. acidophilus* P4	13.00 ± 0.202 ^cd^	14.00 ± 0.404 ^c^
*L. buchneri* P1	8.00 ± 0.289 ^f^	11.00 ± 0.318 ^ef^
*L. brevis* ATCC 8287	9.00 ± 0.058 ^ef^	12.00 ± 0.173 ^de^
*L. salivarius* TISTR 390	0.00	0.00
*B. animalis* subsp. *lactis* (DSMZ)	8.00 ± 0.404 ^f^	7.00 ± 0.144 ^g^

Values represent the mean antifungal activity after 5 days of incubation. Values are presented as mean ± SEM (_n_ = 3). The values represent the diameter of the inhibition zones (mm). SEM was calculated as SD/√*n*. Different lowercase letters within the same column indicate statistically significant differences among LAB strains according to one-way analysis of variance (ANOVA) followed by Tukey’s honestly significant difference (HSD) post hoc test at *p* ≤ 0.05. Values sharing at least one common letter are not significantly different (*p* > 0.05). Inhibition zones were classified as ≤8 mm (low or no antifungal activity), 9–14 mm (sensitive), 15–19 mm (very sensitive), and ≥20 mm (extremely sensitive).

**Table 5:** Effect of *L. acidophilus* ATCC 20552 and *L. plantarum* P3 cell-free supernatants on total damage percentage of wheat grains inoculated with aflatoxin-producing *Aspergillus* species during 30 days of storage.

Fungal Treatment	LAB Treatment	Total Damage (%)							
		*L. acidophilus* ATCC 20552				*L. plantarum* P3			
		0 d *	15 d	30 d	# Mean Damage (%)	0 d	15 d	30 d	Mean Damage (%)
Neg. controls	100% supernatant	4.0	6.0	8.0	6.0	4.0	8.0	9.0	7.0
	50% supernatant	4.0	9.0	21.0	11.33	4.0	11.0	15.0	10.0
	25% supernatant	4.0	12.0	44.0	20.0	4.0	15.0	30.0	16.33
*A. flavus* AF	100% supernatant	4.0	12.0	26.0	14.0	4.0	10.0	17.0	10.33
	50% supernatant	4.0	15.0	28.0	15.67	4.0	12.0	26.0	14.0
	25% supernatant	4.0	24.0	63.0	30.33	4.0	27.0	58.0	29.67
	0% supernatant	4.0	78.0	100.0	60.67	4.0	77.0	100.0	60.33
*A. parasiticus* AP	100% supernatant	4.0	15.0	24	14.33	4.0	13.0	22.0	13.0
	50% supernatant	4.0	23.0	36.0	21.0	4.0	23.0	34.0	20.33
	25% supernatant	4.0	37	73	38.0	4.0	33.0	68.0	35.0
	0% supernatant	4.0	91.0	100.0	65.0	4.0	93.0	100.0	65.67

100% LAB supernatant: original cell-free LAB supernatant. 50% LAB supernatant: cell-free LAB supernatant diluted 1:1 with sterile distilled water. 25% LAB supernatant: cell-free LAB supernatant diluted 1:3 with sterile distilled water. 0% supernatant: wheat grains inoculated with fungal spores without addition of LAB supernatant (pos. control). * Samples collected after 24 h of incubation were designated as the baseline (0 d) measurements. # Values represent the mean total % of grain damage calculated across the storage periods and are presented as descriptive summary values to illustrate the overall damage trend during storage.

**Table 6:** Effect of *L. acidophilus* ATCC 20552 and *L. plantarum* P3 cell-free supernatant on aflatoxin (B_1_, B_2_, G_1_ and G_2_) concentrations in wheat grains inoculated with aflatoxin-producing *Aspergillus* species during storage.

Fungal Treatment	LAB Treatment	AFs Conc. (ppb)									
		*L. plantarum* P3					*L. acidophilus* ATCC 20552				
		B1	B2	G1	G2	Total AFs	B1	B2	G1	G2	Total AFs
Neg. controls	100%	22.0	13.0	9.0	8.0	52.0	21.0	10.0	9.0	0.0	40.0
	50%	1084.0	177.0	446.0	71.6	1778.6	54.0	23.0	17.0	11.0	105.0
	25%	1239.0	236.0	437.0	84.0	1996.0	204.0	29.0	102.0	31.0	366.0
*A. flavus* AF	100%	69.0	8.0	32.0	12.0	121.0	89.0	8.0	31.0	11.0	139.0
	50%	17,442.0	1075.0	2333.0	1511.0	22,361.0	360.0	57.0	116.0	26.0	559.0
	25%	15,484.0	2111.0	2176.0	223.0	19,994.0	3491.0	458.0	5014.0	544.0	9507.0
	0%	39,500.0	20,500.0	8000.0	6260.0	74,260.0	39,900.0	20,100.0	80,100.0	6180.0	146,280.0
*A. parasiticus* AP	100%	58.0	7.0	30.0	7.0	102.0	92.0	14.0	31.0	6.0	143.0
	50%	9717.0	1160.0	4850.0	388.0	16,115.0	8699.0	1951.0	504.0	401.0	11,555.0
	25%	10,000.0	2921.0	3113.0	1270.0	17,304.0	19,486.0	5175.0	489.0	640.0	25,790.0
	0%	21,039.0	6344.0	4456.0	2995.0	34,834.0	21,032.0	6341.0	4455.0	2992.0	34,820.0

100% LAB supernatant: original cell-free LAB supernatant. 50% LAB supernatant: cell-free LAB supernatant diluted 1:1 with sterile distilled water. 25% LAB supernatant: cell-free LAB supernatant diluted 1:3 with sterile distilled water. 0% supernatant: wheat grains inoculated with fungal spores without addition of LAB supernatant (pos. control).

**Table 7:** Protective effect of *L. acidophilus* ATCC 20552 and *L. plantarum* P3 cell-free supernatants on wheat grains by reducing fungal damage and aflatoxin accumulation after 30 days of storage.

Fungal Species	LAB Strain	Reduction of Grain Damage (%)	Reduction of Total Aflatoxins (%)
*A. flavus* AF	*L. plantarum* P3	83.0	99.84
*A. parasiticus* AP	*L. plantarum* P3	78.0	99.71
*A. flavus* AF	*L. acidophilus* ATCC 20552	74.0	99.90
*A. parasiticus* AP	*L. acidophilus* ATCC 20552	76.0	99.59

## Data Availability

The original contributions presented in this study are included in the article.

## Supplementary Materials

The following supporting information can be downloaded at: https://www.mdpi.com/article/10.3390/microorganisms14081710/s1. Figure S1A: HPLC chromatogram of a standard mixture of aflatoxins B_1_, B_2_, G_1_, and G_2_; Figure S1B: Representative HPLC chromatogram of aflatoxins detected in a wheat grain sample.
