# Supplementary material for: Antifungal and Antiaflatoxigenic Potential of Lactic Acid Bacteria Against Aspergillus flavus and Aspergillus parasiticus for Wheat Grain Protection
Source: Microorganisms. 2026 Aug 4;14(8):1710. doi: 10.3390/microorganisms14081710 (PMC13515410; doi:10.3390/microorganisms14081710)
Supplement: Supplementary file 1 [file microorganisms-14-01710-s001.zip › microorganisms-4432510-supplementary.pdf]

Supplementary File

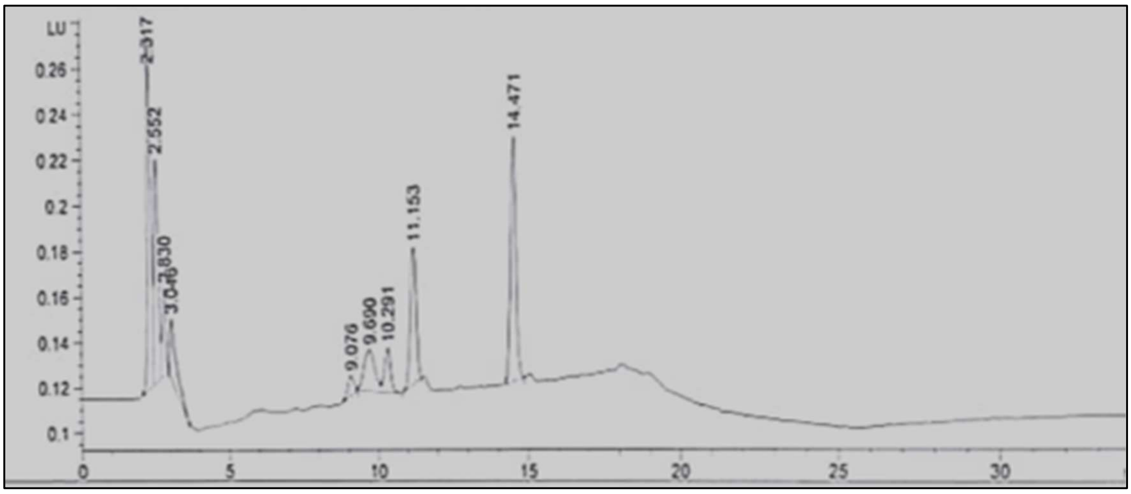

A

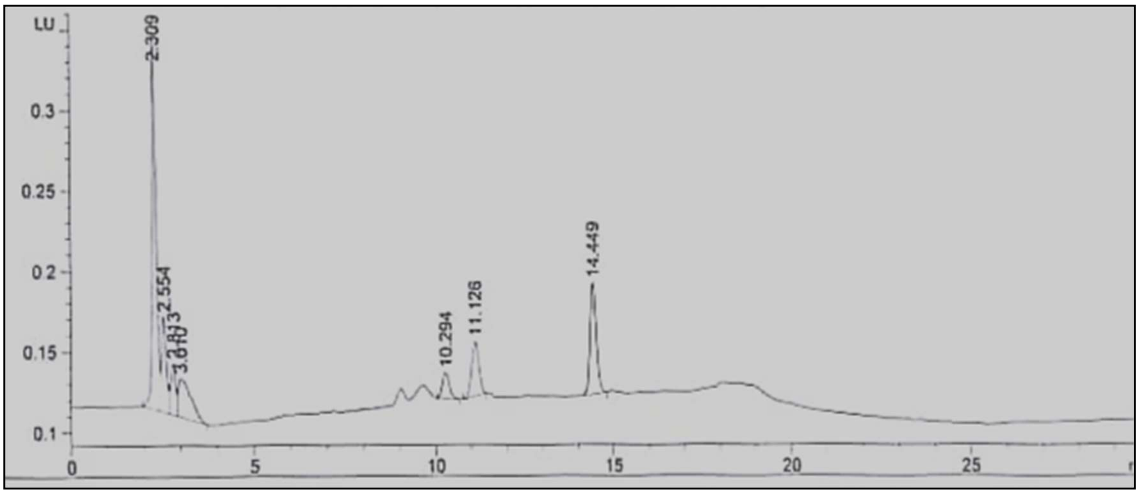

B

Supplementary Figure S1A

HPLC chromatogram of a standard mixture of aflatoxins B_1, B_2, G_1, and G_2

Supplementary Figure S1B

Representative HPLC chromatogram of aflatoxins detected in a wheat grain sample
